# Measuring the geographic coverage of methadone maintenance programme in Hong Kong by using geographic information system (GIS)

**DOI:** 10.1186/1476-072X-7-5

**Published:** 2008-01-30

**Authors:** Tak Ting P Pang, Shui Shan Lee

**Affiliations:** 1 Stanley Ho Centre for Emerging Infectious Diseases, The Chinese University of Hong Kong, Shatin, Hong Kong, China

## Abstract

**Objective:**

While access and utilization form core components in assessing the effectiveness of a health service, the concept of coverage is often neglected. In this study we propose to develop a GIS-based methodological framework for the measurement of district-based geographic coverage to examine the service effectiveness of methadone treatment programme (MTP) in Hong Kong on a regular basis.

**Methods:**

To overcome the incompatibility of spatial units, population data and data of heroin addiction of the year 2001 are interpolated by population-weighted and area-weighted algorithms. Standard overlay and proximity analytical functions are used to delineate altogether 20 accessible zones around each methadone clinic at a fixed 1.5 km Euclidean distance. Geographic coverage here is defined as the percentage of heroin addicts covered by a methadone clinic within the accessible zone by district.

**Results:**

A total of 6413 out of 11000 reported heroin addicts are found geographically covered. The average geographic coverage in Hong Kong is 44.6%, with the figure varying from 0% to 96% by district. One district having no clinic results in 0% coverage whereas another without a clinic yields 15.3% coverage from the clinic in adjacent district. Maps illustrating district-based geographic coverage are generated.

**Conclusion:**

As continuous data collection is required for a monitoring system, the simplified approach facilitates the handling of large volume data and relevant data analysis. It is concluded that the number of methadone clinics is as important as their locations. Geographic coverage could become an important consideration for monitoring harm reduction.

## Background

### Harm reduction and coverage assessment

Methadone maintenance is one of the well-known harm reduction strategies for public health intervention in heroin addiction. The significance of methadone treatment in preventing needle sharing, which in turn reduces the risk of HIV and HCV transmission among injectors, has been well cited. [1-4] Methadone clinic is also considered gathering site where heroin addicts can effectively acquire knowledge on harm reduction and drug rehabilitation.

To achieve maximum effectiveness, methadone clinic should be situated at where it satisfies the needs of local community of heroin users. The identification of the service zone of a health service and an examination of whether it is reachable are crucial. In this connection, the concept of coverage is useful. This is particularly meaningful for evaluating the provision of methadone maintenance programme, a service that delivers public health good. While large proportion of studies to date have focused on access and utilization of health care systems [5-10], few of them discuss the application of coverage, a concept that was initially put forward for over 30 years ago. Recently, World Health Organization (WHO) has developed the HealthMapper and Service Availability Mapping Programme (SAM), which emphasize on visualizing and monitoring the spatial coverage of health care services. Abundant studies have also described the contribution of geographic information systems in public health services management [11-13].

In this study, Hong Kong is taken as an example to pilot the measurement of the district-based geographic coverage of methadone clinics in terms of their availability within defined accessible catchment areas. The study aims at developing a simplified methodological framework to measure the geographic coverage of methadone clinics regularly, which enables a monitoring system to be developed for evaluating harm reduction strategies.

### The geography of Hong Kong

**Figure 1:**
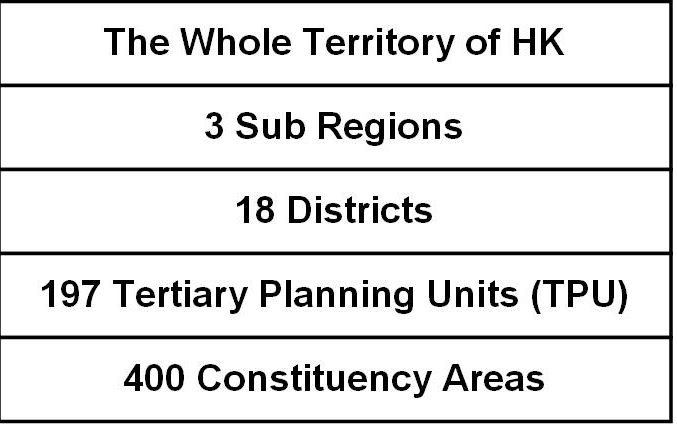
**A table showing levels of geographic units in Hong Kong**. The three sub regions are Hong Kong Island, Kowloon Peninsula and The New Territories. Districts, tertiary planning units and constituency areas are commonly used for census, urban planning and election purposes respectively.

Hong Kong is a metropolitan city located in Southeast Asia, bordering the South China Sea and mainland China [14]. The total land area is about 1100 square kilometres and subdivided into several levels of geographic units. (Figure 1) According to the latest available census data in 2006, there are approximately 6.9 million people in Hong Kong [15]. Heroin is the most prevalent drug abused in the territory. In the year of 2005, 9200 heroin addicts were reported, of which 53% were injecting drug users. Among all drug abusers, 30% of them were multiple drug users [16]. The prevalence of reported addiction was highly skewed into some districts.

### Methadone treatment programme (MTP) in Hong Kong

The MTP has been operated by the Department of Health (previously Medical and Health Services Department) for 30 years. It adopts an out-patient and low threshold approach. Services are provided promptly to all heroin addicts who come forward for methadone treatment. For those with life-threatening medical condition, referral for treatment is made before being started on methadone. Besides prescribing methadone, other related services including patient assessment, counselling services and referral services are provided in methadone clinics [17]. Currently, there are 20 methadone clinics at separate geographic locations with different service hours that are open all year round, regardless of bad weather or public holidays. However, clinics are not distributed evenly in each and every district, whereas heroin addicts are found throughout the territory. The geographic discrepancy between service provision and service demand implies that gap exists, the measurement of which would contribute to an assessment of coverage for those in need.

## Methods

### Data Source

Essential data for this study included population data and data of heroin addiction. All data obtained were of the year 2001, when consistent data types from different services could be assured. As a study to demonstrate the methodological framework of coverage measurement, the use of the 2001 data, instead of more updated but inconsistent datasets, would be more important to enable reliable calculations to be made. District-based and TPU-based population data were collected from the Census and Statistics Department, while district-based data of heroin addiction was acquired from the Central Registry of Drug Abuse (CRDA), Narcotics Division of Security Bureau. CRDA has been established for 35 years to capture drug abuse data, which are provided by the reporting agencies on a voluntary basis with their full cooperation. The statistics is used to monitor the trend of drug abuse and to facilitate the planning of drug rehabilitation programmes in Hong Kong.

Digital base maps including the boundary map of districts, boundary map of TPUs, map of highlands and water bodies were obtained from the Survey and Mapping Office (SMO), Lands Department of Hong Kong Government. Locations of methadone clinics were stored as X, Y projected coordinates in the GeoCommunity Database table (Version 2.1.2), which was also acquired from the SMO. These data, after synchronization, were input into a GIS software, ArcGIS 9.1 [18], for data processing.

### Assumptions for refining available data

The landscape in Hong Kong is mountainous. About one-fifth of land area is over 200 metres in altitude, which is defined as uninhabitable highland in this study. Furthermore, the result will be highly unreliable if heroin addicts are assumed to distribute over the entire district, considering that only part of the district is populated. District-based heroin addicts' data were apportioned into TPUs by areal interpolation before measuring district-based geographic coverage. From the interactions with current and former heroin users, three assumptions for this study were made. First, walking is a preferred means of going to methadone clinics. Second, heroin addiction is assumed to be rather localized. Heroin addicts seldom go to the methadone clinic that is not located in their residence district. Third, heroin addicts are willing to walk at most for 30 minutes to their local methadone clinics.

### Elimination of uninhabitable area

**Figure 2:**
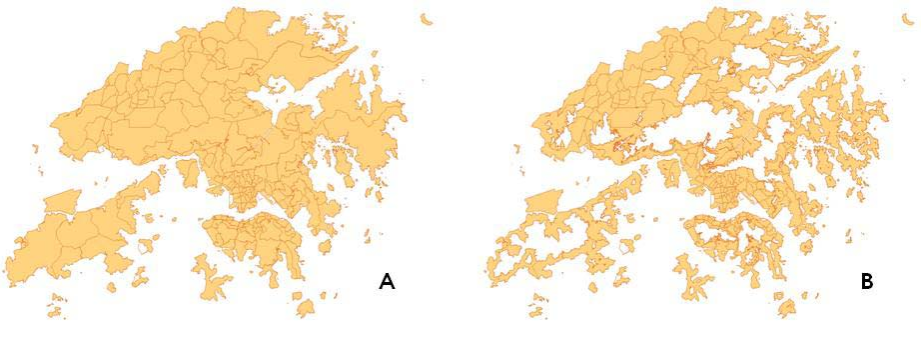
A map showing (A) Whole territory of Hong Kong; (B) Inhabitable areas after elimination of highland and water bodies indicated as hollow areas.

In preparing the Hong Kong base maps for spatial analysis, all water bodies, including reservoir and rivers, and land elevated over 200 metres were eliminated. (Figure 2) As there was slight difference between the outline boundary of district and TPU, district base map was used as a reference to adjust TPU boundary. Population data were joined to the attribute tables of the base maps of district and TPU. As a result, a TPU-based boundary map of inhabitable area connected with population data was generated.

### Areal interpolation

Incompatibility of different geography/geometry of spatial units impedes spatial analysis, especially when most available health data are aggregated into different geographic units, which has been addressed as polygon overlay problem [19]. A typical solution to the problem is to use areal interpolation, which refers to the process of transforming data (the source layer) with one zoning system to another zoning system (the target layer), based on defined algorithms [20]. There are different algorithms, such as area-weighted, population-weighted, street-weighted, which should be used according to the nature of data [21]. However these algorithms share one basic assumption, that spatial objects in the source zone are distributed uniformly. In this study, population- and area- weighted algorithm are applied under two different situations.

**Figure 3:**
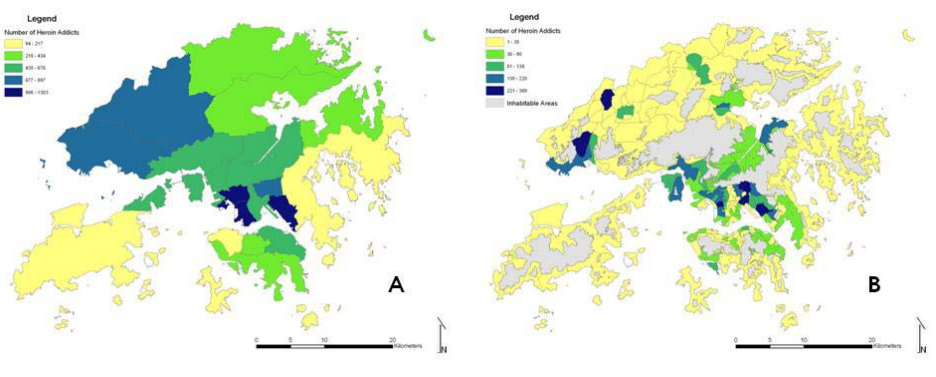
**Spatial distribution of heroin addicts over (A) Districts; (B) TPUs**. The two maps show the difference in spatial distribution of heroin addicts before and after population weighted areal interpolation. It is obvious that the TPU map presents better local variations of addiction pattern while district presents a rather raw picture of the addicts' distribution.

Zones of TPU were not completely contained in a specific district. By intersecting the base maps, the corresponding district of each TPU was determined based on the location of its centroid. Then, population-weighted areal interpolation was carried out to transform district-based heroin data into TPU-based. It was assumed that the number of heroin addicts by TPU (target layer) is proportional to its population, sharing the same ratio of heroin addicts by district over population by district. (Figure 3) Thus the new dataset of TPU-based heroin addicts was generated by:

Number of heroin addicts by district / population by district * population by a specific TPU

**Figure 4:**
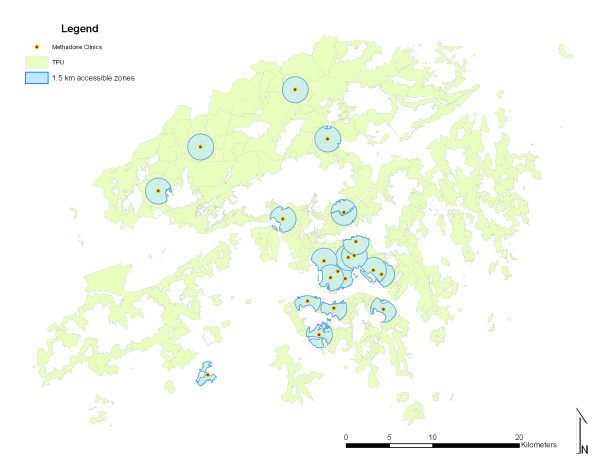
Accessible zones of 1.5 km of each clinic after eliminating uninhabitable areas.

Regarding the assumption of maximum walking time of 30 minutes, a buffer of 1.5 Kilometre *Euclidean *distance was defined as accessible zone for each clinic, assuming that addicts will cover this distance in 30 minutes. (Figure 4)

The availability, that is the number of addicts covered by a methadone clinic within accessible zones, was calculated by area-weighted areal interpolation. It was hypothesized that the area covered by the accessible zones was that accessed by addicts within the accessible zones in each TPU as well, which shared the same ratio of addicts in a TPU over the total area of the entire TPU. TPUs that intersected with accessible zones were picked up. Visual Basic Application (VBA) statements were used in the GIS to calculate both the area of entire intersected TPUs and the area of intersected portion of TPU. Percentage of area covered by the accessible zones in each intersected TPU was calculated and TPU-based heroin addicts covered were estimated by using the percentage. The following equation was used:

Area of a TPU within the accessible zone / Area of the entire corresponding TPU* Interpolated number of heroin addicts in that TPU

### Measurement of district-based geographic coverage

Number of addicts from all TPUs in a district covered within accessible zones was aggregated into their corresponding district. However, the number of methadone clinics in each district varied considerably. The number of district-based addicts covered was adjusted and the mean number of heroin addicts covered by one methadone clinic by district was calculated. District-based geographic coverage was expressed as the percentage of heroin addicts covered within those accessible zones in each district among all heroin addicts in that district, using the following formula:

Number of heroin addicts covered within a accessible zone in a district / Total number of heroin addicts in that corresponding district* 100%

## Results

In the year 2001, there were about 11000 reported heroin addicts, giving a prevalence of 166 heroin addicts per 100,000 population in Hong Kong. The prevalence in eight out of 18 districts was higher than the average, with Yau Tsim Mong yielding the highest rate of 403. The distribution of methadone clinic was uneven. While one district was having three methadone clinics, two districts including Kwai Tsing and Sai Kung did not have any. Most of the districts (13 out of 18) had one methadone clinic, with two districts having two. After the elimination of uninhabitable areas, all TPUs (N = 197) remained populated and were therefore valid for conducting areal interpolation. The population-weighted areal interpolation produced interpolated values of heroin addicts by TPUs ranging from 1 to 369, with one-forth of TPUs (50/197) having less than 10 heroin addicts. Though all TPUs could be subjected to population-weighted areal interpolation, only 125 of them gave an intersection with the defined accessible zones for a clinic. In fact, an average of 66% of TPUs in a district was covered by the accessible zones. Accessible zones in four districts even covered all TPUs in that particular district.

**Figure 5:**
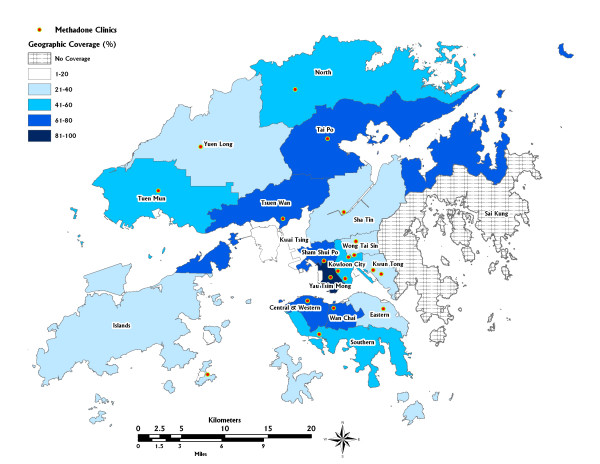
Map showing geographic coverage of methadone clinics in 18 districts 2001. Sai Kung is the only district with no coverage (the patterned district) while Yau Tsim Mong has the highest coverage in Hong Kong (district with the darkest colour).

Using the definition developed in this study, a total of 6413 of 11000 heroin addicts were geographically covered by all methadone clinics in 2001. Since there were more than one methadone clinic in some districts, adjusted number of heroin addicts covered in each district was calculated. Only one district, Sai Kung, showed no coverage by any methadone clinic. Geographic coverage varied from the lowest 15.3% in Kwai Tsing to the highest figure 96.2% in Yau Tsim Mong. In the remaining districts, the average geographic coverage was 44.6% in 2001, though eight out of 18 districts demonstrated over 50% geographic coverage. (Table 1) A map was generated to show the final result of district based geographic coverage in Hong Kong (Figure 5).

**Table 1:** District-based geographic coverage 2001. The table shows the geographic coverage of all 18 districts in Hong Kong, with reference to the adjusted number of heroin addicts covered according to the number of methadone clinics in each district.

**District**	**Heroin addicts covered**	**No of methadone clinic**	**Adjusted No of heroin addicts covered**	**Total No of heroin addicts**	**Geographic coverage (%)**
**Sai Kung**	0	0	0	217	0.0
**Kwai Tsing**	96	0	96	626	15.3
**Wong Tai Sin**	431	2	216	997	21.6
**Islands**	21	1	21	94	22.3
**Yuen Long**	215	1	215	775	27.7
**Sha Tin**	154	1	154	541	28.5
**Eastern**	236	1	236	676	34.9
**Kwun Tong**	833	2	417	1076	38.7
**Tuen Mun**	364	1	364	908	40.1
**North**	186	1	186	434	42.9
**Kowloon City**	844	3	281	557	50.5
**Southern**	168	1	168	327	51.4
**Wan Chai**	173	1	173	287	60.3
**Tsuen Wan**	351	1	351	581	60.4
**Shum Shui Po**	801	1	801	1303	61.5
**Tai Po**	281	1	281	385	73.0
**Central & Western**	166	1	166	215	77.2
**Yau Tsim Mong**	1093	1	1093	1136	96.2

**Total**	6413	20	5219	11135	44.6 (Average by district)

## Discussion

### Evolving definition of coverage

The idea of coverage has emerged from the concern of community resources allocation and thus has led to the development of health service indicators in late 1960s. However, attention on coverage soon turned towards access and utilization, which reflected a more concrete principle to justify resource investment for policy makers [22]. In 1978, Tanahashi of WHO noticed and suggested a major reason for the failure of "coverage" in drawing people's attention

*"...the confusion about the concept of coverage which has been measured and interpreted differently for various purposes and occasions deter one's incentive to use." *[23]

Subsequently in an evaluation report on health performance from WHO, coverage assessment was identified as playing an instrumental role to evaluate intermediate goals of health services. Five domains of coverage were then delineated, namely availability coverage, accessibility coverage, acceptability coverage, contact coverage and effective coverage [22]. Each of the five coverages had a core definition and they varied according to their contextual meanings. In this study, geographic coverage was specifically defined in the context of the availability and accessibility of methadone maintenance treatment service in a spatial context.

In this study, accessibility was considered before calculating the availability. Accessibility of health care is a measure of the proportion of a population that reaches appropriate health services [24]. It is multidimensional as there are numerous factors affecting the degree to reach a service [25]. WHO has suggested three types of accessibility, viz., financial accessibility, geographical accessibility and cultural accessibility. However, only geographical accessibility has been taken into account in this study. Financial accessibility has not been considered in our Hong Kong study as the service is directly funded by the Government. All methadone users are required to pay a nominal HKD 1 (HKD 7.8 = USD 1) only for methadone treatment, resulting in almost no financial barrier in accessing MTP. On the other hand, cultural accessibility refers to whether the health service has violated local taboo. The MTP has been set up for over 30 years and is in contact with almost all heroin addicts. The relevance of accessibility discrepancy due to cultural reason is therefore considered to be minimal.

The concept of availability is much simpler than accessibility. It basically denotes the presence or absence of essential health services to the population in need in specific regions [26]. The objective of most studies on availability is often related to the examination of insufficiency of health care service in rural regions and urban deprived areas. Some studies even measured the quality of services to reflect availability. In general, availability refers to the presence or absence of needed health care services [24] and is often expressed as a ratio between the number of health facilities per spatial unit, number of people or unit area [27]. Considering that there is no preset capacity limit for methadone clinics in Hong Kong, their availability in a specific district is measured as the number of heroin addicts covered by the service within a predefined accessible zone over the number of addicts in the corresponding district.

### A simplified approach for coverage monitoring

Since the idea of coverage is still vague, it is almost impossible to employ a standard approach in its measurement. A standard definition is desirable for scientific investigation, but is less likely to be practical. In public health practice, the approach to coverage assessment shall be shaped by the objective. When assessing coverage for monitoring purposes, in contrast to a communication tool for reporting figures at one point of time, a simplified approach is essential to facilitate continuous collection of data on service operation [22]. There is currently no universal coverage measurement of any health service system as it varies according to interventions. In this study, within the analytical framework of coverage, concepts of accessibility and availability were considered, giving us the result of coverage which is solely geographic.

The simplified approach in this study has focused on measurement of coverage using minimum amount of the most determining variables. Neither personal data nor geocoding process was involved in this study. All the data used in this study were from open-source, either downloaded from official websites or extracted from formal reports. The geospatial data of Hong Kong were purchased from the Lands Department. Adoption of available user-friendly technology does also contribute to the simplified coverage assessment system. The geographic information system used in this study was a commercial product. All the functions applied were standard overlay and proximity analytical functions of the software. These functions could be linked up by Visual Basic scripts so that a standard protocol can be developed to activate the whole monitoring system. The study demonstrated how existing resources can be mobilized to build a coverage monitoring system for the MTP in Hong Kong, which can also be adapted for use in other countries. With the scaling up of harm reduction efforts in many countries, the system could be turned into a standard tool for coverage assessment.

### Interpretation of coverage information

Several observations could be made in this study. It is obvious that district-based geographic coverage has varied among districts in Hong Kong. The distinctive pattern is believed to be at least partly related to the size of districts. Districts having over 50% geographic coverage are mainly located on both sides of the Victoria harbour. They are usually smaller in size, more densely populated and are more urbanized compared to the other districts. Buffer based on Euclidean distance tends to capture more people in densely populated than sparsely populated zones. It does not however mean that only addicts within the accessible zones can reach the methadone clinic. Actually, over 95% of heroin addicts in Hong Kong are in contact with the methadone clinics. [28] The geographic coverage in the study is a standardized approach for addressing the percentage of heroin addicts that can be in contact with the service in the local community.

It is noted that most research in assessing health care services are often nation-wide. They may ignore the neighbourhood variations within the country. A district-based approach to evaluate the MTP coverage in Hong Kong served as an example of how unique local patterns can be elicited. The two districts, Kwai Tsing and Sai Kung, without methadone clinic gave a different pattern of geographic coverage. (Table 1) In Kwai Tsing, about 16% of its heroin addicts were covered by the methadone clinic located in adjacent district, Tsuen Wan, within the accessible zone. Surprisingly, the geographic coverage in Tsuen Wan was quite satisfactory, seemingly not being affected by sharing service to Kwai Tsing district. On the other hand Sai Kung is the only district that does not have a methadone clinic and has 0% geographic coverage.

Based on the primary observations of the geographic coverage of MTP, resources and attention could be directed to those districts with less satisfactory geographic coverage. It can be concluded in this study that the number of clinics is as important as their locations, evidenced by the districts without methadone clinic. Distinguishing the spatial discrepancy between the location of methadone clinic and heroin addicts is one of the effective means to have the methadone system improved. Furthermore, some underlying factors such as immigrant population, social economic status of a population and the capacity of methadone clinics may be associated with the skewed pattern of geographic coverage. Thus identification of districts of low geographic coverage might help uncovering the linkage between methadone clinics and social phenomenon.

The present study has demonstrated how the application of GIS, together with concept of geographic coverage, helps developing a standard tool to inform and empower decision making for service management of harm reduction programmes [29]. However, the methodology introduced here should be considered in light of the following limitations. First, this study fails to reveal the actual coverage of methadone clinics. The total coverage of each methadone clinic should be higher since the whole methadone programme covered over 95% of heroin addicts in Hong Kong. [28] While considering geographic proximity is the most influential factor in affecting the incentive to use the service, we admit that other factors, such as the fluctuation of opium price/availability in black market or the severity of need, do contribute to the total coverage of methadone clinic. Second, the assumptions listed in the study were made only based on broad images suggested by several rehabilitated addicts. Further concrete surveying of addicts' utilization pattern should be required, which provides more explicit results for assumption building. Finally, different methods to measure accessibility have been discussed by researchers. By comparing network-based distance and Euclidean distance, some researchers believed that Euclidean distance reflects false representation of distance whereas some thought that the network-based distance offered no better result than Euclidean distance [30-32]. In this study Euclidean distance was used because of two further considerations. While the cost in obtaining road network data is high and restricted, the up-datedness of data is another key concern. Since Hong Kong is a densely populated city, together with an extensive pedestrian road network, the walking distance is not very much different from the straight line distance between two locations. On top of the limitations, the interpretation of coverage information should be considered with caution, as it is highly dependent on the completeness of one single data source, CRDA

## Conclusion

The establishment of a coverage assessment system is not intended to highlight the best measurement of coverage, but to demonstrate a framework to capture the coverage information indicating whether a region is well supplied in a spatial perspective and enable policy makers to ensure satisfactory coverage to the need [32]. In this study, we only focus on assessing the geographic coverage which is believed to be the prominent factor that policy makers have to take into account for future MTP development. In the future, more aspect of coverage, such as effective coverage suggested by WHO, should also be under examination to advance the monitoring system.

We expect to see that GIS, together with the re-emerging concept of coverage, would provide new insights to address policy concerns of health service planning, as the use of spatial technologies is especially conspicuous in assessing non-quantifiable variables such as ethnicity and culture, recognising the importance of qualitative approach to address health service delivery matters.

## Competing interests

The author(s) declare that they have no competing interests.

## Authors' contributions

SSL was involved in the conceptualization and research design of the study. PTTP conducted the cartographic design and GIS analyses. This manuscript was drafted by PTTP and edited by SSL. Both authors read and approved the final manuscript.
